# Ventricular tachycardia ablation associated abnormalities on FDG PET/CT in patients with suspected cardiac sarcoidosis

**DOI:** 10.1093/europace/euag050

**Published:** 2026-03-14

**Authors:** Brigitte Kazzi, Stephen J Hankinson, Sylvain L Carre, Jon Hainer, Akshay S Desai, Garrick C Stewart, Neal K Lakdawala, Michael M Givertz, Ron Blankstein, Marcelo F Di Carli, Bruce A Koplan, Usha B Tedrow, William H Sauer, Sanjay Divakaran

**Affiliations:** Cardiovascular Imaging Program, Departments of Medicine and Radiology, Brigham and Women’s Hospital, 75 Francis Street, Boston, MA 02155, USA; Division of Cardiovascular Medicine, Department of Medicine, Brigham and Women’s Hospital, Boston, MA, USA; Harvard Medical School, Boston, MA, USA; Cardiovascular Imaging Program, Departments of Medicine and Radiology, Brigham and Women’s Hospital, 75 Francis Street, Boston, MA 02155, USA; Division of Cardiology, Department of Medicine, Rutgers Robert Wood Johnson Medical School, New Brunswick, NJ, USA; Cardiovascular Imaging Program, Departments of Medicine and Radiology, Brigham and Women’s Hospital, 75 Francis Street, Boston, MA 02155, USA; Cardiovascular Imaging Program, Departments of Medicine and Radiology, Brigham and Women’s Hospital, 75 Francis Street, Boston, MA 02155, USA; Division of Cardiovascular Medicine, Department of Medicine, Brigham and Women’s Hospital, Boston, MA, USA; Harvard Medical School, Boston, MA, USA; Division of Cardiovascular Medicine, Department of Medicine, Brigham and Women’s Hospital, Boston, MA, USA; Harvard Medical School, Boston, MA, USA; Division of Cardiovascular Medicine, Department of Medicine, Brigham and Women’s Hospital, Boston, MA, USA; Harvard Medical School, Boston, MA, USA; Division of Cardiovascular Medicine, Department of Medicine, Brigham and Women’s Hospital, Boston, MA, USA; Harvard Medical School, Boston, MA, USA; Cardiovascular Imaging Program, Departments of Medicine and Radiology, Brigham and Women’s Hospital, 75 Francis Street, Boston, MA 02155, USA; Division of Cardiovascular Medicine, Department of Medicine, Brigham and Women’s Hospital, Boston, MA, USA; Harvard Medical School, Boston, MA, USA; Cardiovascular Imaging Program, Departments of Medicine and Radiology, Brigham and Women’s Hospital, 75 Francis Street, Boston, MA 02155, USA; Division of Cardiovascular Medicine, Department of Medicine, Brigham and Women’s Hospital, Boston, MA, USA; Harvard Medical School, Boston, MA, USA; Division of Cardiovascular Medicine, Department of Medicine, Brigham and Women’s Hospital, Boston, MA, USA; Harvard Medical School, Boston, MA, USA; Division of Cardiovascular Medicine, Department of Medicine, Brigham and Women’s Hospital, Boston, MA, USA; Harvard Medical School, Boston, MA, USA; Division of Cardiovascular Medicine, Department of Medicine, Brigham and Women’s Hospital, Boston, MA, USA; Harvard Medical School, Boston, MA, USA; Cardiovascular Imaging Program, Departments of Medicine and Radiology, Brigham and Women’s Hospital, 75 Francis Street, Boston, MA 02155, USA; Division of Cardiovascular Medicine, Department of Medicine, Brigham and Women’s Hospital, Boston, MA, USA; Harvard Medical School, Boston, MA, USA

**Keywords:** FDG PET/CT, Ablation, Ventricular tachycardia, Perfusion–metabolic mismatch, Cardiac sarcoidosis

## Introduction

Clinical hallmarks of cardiac sarcoidosis (CS) include left ventricular systolic dysfunction, high-grade atrioventricular block, and ventricular arrhythmias.^1^ Fluorodeoxyglucose (FDG) positron emission tomography/computed tomography (PET/CT) imaging can help diagnose CS and monitor response to therapy.^1^ Myocardial perfusion–metabolic mismatch on FDG PET/CT is a specific imaging finding for CS in the absence of obstructive coronary artery disease.^1^

Recurrent ventricular tachycardia (VT) is common in CS and greatly impacts quality of life and prognosis. Studies have demonstrated that, among those with recurrent arrhythmia or who do not tolerate antiarrhythmic drugs, catheter ablation effectively decreases arrhythmic burden.^2^ Ablation studies in patients with CS frequently demonstrate a complex myocardial substrate, even without active inflammation, which can involve the Purkinje system, both ventricles, and intramural or epicardial locations.^1^ In the oncology literature, the challenges of attributing FDG uptake soon after ablative treatment to residual disease vs. ablation-related inflammation are recognized.^3,4^ The analogy in cardiology would be mistaking ablation-related abnormalities for signs of inflammatory cardiomyopathy. In this study, we aimed to evaluate imaging findings of patients who underwent ablation prior to FDG PET/CT for possible CS with a particular focus on the prevalence and timing of perfusion–metabolic mismatch.

## Methods

This retrospective cohort included patients referred to our centre for FDG PET/CT for suspected CS from June 2006 to November 2023^5^ who underwent ventricular catheter ablation within 365 days before the FDG PET/CT. The Mass General Brigham Institutional Review Board approved this study.

All patients were instructed to follow at least two high-fat, very low-carbohydrate meals prior to FDG PET/CT imaging. Perfusion images were classified as normal or abnormal (summed rest score > 2). Myocardial FDG images were classified as no focal myocardial FDG uptake, focal myocardial FDG uptake, or non-specific myocardial FDG uptake. Myocardial FDG uptake was quantified using previously described methods.^6^ An SUV threshold of 2.7 was used to quantify cardiac metabolic volume (CMV).^7^ Perfusion–metabolic mismatch (abnormal perfusion associated with focal FDG uptake) was identified and recorded. Extracardiac FDG images were classified by the presence or absence of FDG uptake compatible with metabolically active extracardiac sarcoidosis.

Patients were categorized into three diagnostic groups based on clinical evaluation and imaging findings: (1) Heart Rhythm Society (HRS) criteria^8^ positive CS or probable CS (extracardiac FDG uptake and HRS criteria for CS except biopsy-proven disease); (2) ischaemic cardiomyopathy (CMP); or (3) all other diagnoses, including idiopathic CMP, myocarditis, genetic CMP, and non-ischaemic CMP.

Categorical variables were presented as counts (percentages) and compared using the χ^2^ or Fisher’s exact test as appropriate. All analyses were conducted using Stata version 13 (StataCorp, College Station, TX).

## Results

We identified 104 patients who met inclusion criteria. The cohort consisted of 17 patients (16%) with HRS positive or probable CS, 14 patients (14%) with ischaemic CMP, and 73 patients (70%) with other diagnoses. The median interval between VT ablation and PET/CT was 11.5 days (2.0–68.3). Fifty-eight patients (56%) underwent FDG PET/CT ≤ 30 days after VT ablation [early, median 2 days (1–4)], and 46 patients (44%) underwent FDG PET/CT > 30 days and ≤ 365 days after VT ablation [late, median 71 days (51–113)].

**Figure 1:**
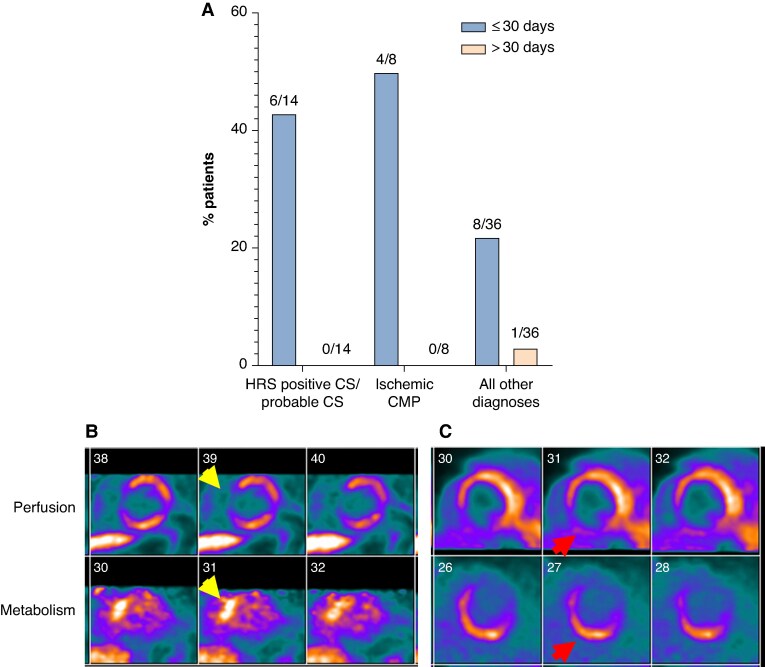
Per cent of patients with perfusion–metabolic mismatch by diagnosis and timing between VT ablation and fluorodeoxyglucose positron emission tomography/computed tomography and representative perfusion–metabolic mismatch findings early after ventricular tachycardia ablation. (*A*) Frequency of perfusion–metabolic mismatch by clinical diagnosis and timing between ventricular tachycardia ablation and imaging. (*B*) Perfusion–metabolic mismatch involving the basal anteroseptal wall (yellow arrows) in a patient with other diagnosis. Ablation of the left ventricular outflow tract was performed 3 days prior. (*C*) Perfusion–metabolic mismatch involving the basal inferoseptal and inferior walls (red arrows) in a patient with cardiac sarcoidosis. Ablation of the inferior wall and the septum was performed 2 days prior. CMP = cardiomyopathy. CS = cardiac sarcoidosis. HRS = Heart Rhythm Society.

Among the 58 patients who underwent FDG PET/CT early post-ablation, abnormal myocardial perfusion was observed in 10 of 14 patients (71%) with CS, 5 of 8 (63%) with ischaemic CMP, and 18 of 36 (50%) with other diagnoses (*P* = 0.37). There were no differences in rates of focal FDG uptake (43% vs. 38% vs. 39%, respectively, *P* = 0.17). Perfusion–metabolic mismatch was present in 6 of 14 patients (43%) with CS, 4 of 8 (50%) with ischaemic CMP, and 8 of 36 (22%) with other diagnoses (*P* = 0.17, ***Figure 1***).

Among patients who underwent imaging late after VT ablation, abnormal myocardial perfusion was observed in 2 of 3 (67%) of the CS group, 4 of 6 (67%) of the ischaemic CMP group, and 11 of 36 (30%) of the other diagnoses group (*P* = 0.12). Most patients demonstrated no focal FDG uptake [3 of 3 (100%), 5 of 6 (83%), and 27 of 36 (73%), respectively; *P* = 0.52]. Late imaging perfusion–metabolic mismatch was absent in both CS and ischaemic CMP groups and present in only one patient with other diagnosis (***Figure 1***).

Across all patients with quantifiable myocardial FDG uptake, mean myocardial SUV_max_ (6.57 ± 1.72 vs. 6.00 ± 2.77, *P* = 0.60) and CMV (185.7 ± 94.0 mL vs. 82.5 ± 107.8 mL, *P* = 0.002) were higher within 30 days after ablation compared to beyond 30 days. The location of VT ablation correlated with the area of perfusion–metabolic mismatch in 16 of 19 (84.2%) patients.

Three of the 19 total patients who had perfusion–metabolic mismatch present underwent follow-up FDG PET/CT 207 ± 86 days after the initial study (individual follow-up durations were 110, 238, and 272 days). All three had CS and all three were treated with immunosuppression between studies. Perfusion–metabolic mismatch was present in two of the three follow-up studies. Abnormal FDG uptake was present on follow-up imaging in four of seven patients. VT recurrence occurred in one of these four patients, compared with two of three patients without abnormal FDG uptake on follow-up (*P* = 0.49)

## Discussion

Across the cohort, perfusion–metabolic mismatch was most frequent early after VT ablation and largely absent in late imaging, irrespective of underlying diagnosis. Our findings suggest that prevalence of perfusion–metabolic mismatch declines after the first month post-VT ablation. These temporal dynamics are consistent with a study by Ohira et al. of a rabbit model of lung radiofrequency ablation, in which FDG uptake around ablation lesions declined substantially by 4–8 weeks (3). Our findings support a cautious approach when using FDG PET/CT imaging for the diagnosis of inflammatory CMP or assessment of disease activity in patients who recently underwent VT ablation. Importantly, in standard clinical practice, FDG PET/CT imaging and the treatment of active myocardial inflammation should occur prior to catheter ablation whenever possible.

Limitations of our study include single-centre, retrospective, cross-sectional design with relatively small sample sizes in some categories and selection bias. FDG PET/CT was not available pre-ablation or serially, data on immunosuppressive therapy and histopathologic validation of inflammation were not available, and only three patients who had perfusion–metabolic mismatch after VT ablation underwent follow-up FDG PET/CT imaging. Future prospective studies with pre- and post-ablation as well as serial imaging could better define the natural evolution of ablation-related FDG uptake.

In conclusion, we found that FDG PET/CT performed within 30 days of VT ablation frequently demonstrated perfusion–metabolic mismatch, irrespective of underlying diagnosis. Our findings suggest that clinicians should consider delaying FDG PET/CT imaging for at least 30 days post-VT ablation for the assessment of suspected CS.

## Data Availability

The data that support the findings of this study are available from the corresponding author, SD, upon reasonable request.
